# Determination of Essential and Toxic Elements in Cattle Blood: Serum vs Plasma

**DOI:** 10.3390/ani9070465

**Published:** 2019-07-21

**Authors:** Diego Luna, Marta López-Alonso, Yolanda Cedeño, Lucas Rigueira, Víctor Pereira, Marta Miranda

**Affiliations:** 1 Department of Animal Pathology, Veterinary Faculty, Universidade de Santiago de Compostela, 27002 Lugo, Spain; 2 Faculty of Veterinary Medicine, Universidad Central del Ecuador, EC170521 Quito, Ecuador; 3 Department of Anatomy, Animal Production and Clinical Veterinary Sciences, Veterinary Faculty, Universidade de Santiago de Compostela, 27002 Lugo, Spain

**Keywords:** trace and toxic elements, serum, plasma, cattle, ICP-MS

## Abstract

**Simple Summary:**

Knowledge of type of blood sample (serum or plasma) for determination of essential and toxic elements is essential for assessing the health and nutritional status of the animals and the herd. We demonstrate that both plasma and serum samples are suitable and interchangeable for the determination of most of the essential and toxic elements in blood in cattle. Nevertheless, we must take into account that the concentrations of Cu and Se are lower in the serum than in plasma.

**Abstract:**

This study was designed to evaluate the influence of type of blood sample (serum or plasma) on essential and toxic element analysis in cattle. Paired plasma and serum samples (*n* = 20) were acid digested, and the concentrations of As, B, Ba, Ca, Cd, Co, Cr, Cu, Fe, Hg, Li, Mg, Mn. Mo, Ni, P, Pb, Sb, Se, Sr and Zn were determined by inductively coupled plasma mass spectrometry (ICP-MS). The study findings indicate that plasma and serum samples appear suitable and interchangeable for the determination of most of the essential and toxic elements in blood in cattle. The only exceptions are Cu and Se, the concentrations of which were significantly lower (40.9 and 29.9% respectively) in serum than in plasma. Some of the Cu in blood samples from bovine ruminants is known to be sequestered during clotting. However, further research on Se in ruminants and other animal species is warranted. Finally, the significantly higher Mn (9.9%) concentrations in serum than in plasma may have been caused by haemolysis of some samples. Special attention should be paid to preventing haemolysis of samples during collection and processing, in order to prevent overestimation of elements present at high concentrations inside erythrocytes (i.e., Fe, Mn and Zn).

## 1. Introduction

Two centuries after the discovery that I and Fe are essential elements, trace element nutrition in humans and livestock animals remains a challenge. Nowadays, at least 17 trace elements are known to be essential for proper growth, health and reproduction [1]. Correct trace mineral status is essential to ensure maximum production in farm animals. Exposure of livestock to anthropogenic and natural sources of toxic elements is also of great concern, as animal products represent one of the main sources of toxic elements in human diets [2]. 

Trace element disorders have been described in many parts of the world, mainly in relation to their geological origin [1]. They continue to appear when new production methods in which soil plays an important part, i.e., organic and other sustainable systems, are used [3]. Although trace element imbalances are known to be related to clinical deficiencies (and less frequently toxicities), subclinical disorders that involve complex interactions between elements are nowadays more relevant [4]. Moreover, although it was traditionally believed that trace elements could be supplied with wide safety margins, it is now known that optimal trace element requirements occur within a narrow range and deviations can have pronounced effects on overall animal health [5]. 

Determining trace mineral status and toxic element accumulation in farm animals provides useful information for veterinary practitioners and is a critical part of maintaining herd health [5]. Trace element analysis was previously complicated as it was very time consuming and inaccurate for some elements present in very low concentrations in blood (e.g., Mo and Co). However, modern and multielement inductively coupled plasma mass spectrometry (ICP-MS) techniques, together with the development of sample preparation protocols that prevent matrix-related interference [6], enable the accurate, rapid and relatively inexpensive routine determination of most trace elements by analysis of a single blood sample in multidisciplinary laboratories. 

An important factor that often is not taken into account is the type of the sample analysed. Whole blood (collected in ethylenediamine tetraacetic acid (EDTA), heparin or other anticoagulants) is scarcely used, but there is little information about the convenience of using serum or plasma samples, or whether the results are comparable. The main difference between serum and plasma is that fibrinogen and clotting factors are consumed in the former, although many other proteins, water, salts and blood cells are caught between the mesh of cross-linked fibrin protein [7]. In relation to trace element analysis, the matrix and composition of serum and plasma are different as some elements bound to proteins can be trapped during clotting. For example, in ruminants, serum concentrations of Cu are 10–30% lower than plasma concentrations, as the Cu-carrying blood protein ceruloplasmin can be sequestrated during clotting [8,9,10]. 

Information about the effect of clotting-related sequestration on other essential or toxic elements, as well as the possible influence of the blood sample matrix (serum vs plasma) during ICP-MS analysis of samples is scarce, although it may be very important. This study evaluates the influence of type of blood sample (serum vs plasma) on essential and toxic element analysis in cattle. 

## 2. Material and Methods

Cows were managed according to Directive 2010/63/EU on the protection of animals used for scientific purposes and the trial complied with the Spanish legislation on animal care (RD 53/2013, 1 February 2013). The procedures applied were supervised by the Bioethics Committee of the University of Santiago de Compostela (Lugo, Spain).

### 2.1. Samples

The bovine blood samples (*n* = 20) used in this study were obtained as part of a wider on-going project on mineral nutrition in cattle. Briefly, the study was conducted in June of 2015 in a commercial dairy farm located in the Province of Lugo (Galicia-Spain) (latitude: 43.0625, longitude: −7.8208). The farm is typical of conventional dairy farms in North Spain, with a loose housed system with slatted floor, beds with sand and a tie-stall system for feeding. It has 236 Holstein Friesian cows in lactation with a 305-d-corrected milk yield of 11.142 kg. The animals were kept indoors and fed total mixed rations (TMR) based mainly on maize silage, hay silage and concentrate with a standard mineral supplementation for lactation, to cover the animal requirements [11]. For this study 20 of the 188 Holstein Friesian lactating cows of more than 2 lactations were selected to obtain a homogenous group (all animals sampled were of 2 or 3 lactation and were between 60–90 days in milk). For each cow, 2 types of blood sample were collected (in triplicate). To obtain plasma, whole blood was collected in 9 mL sodium heparin tubes (Vacuette^®^, NH Trace Elements, Greiner bio-one, Kremsmünster, Austria), whereas serum samples were fractionated from whole blood samples collected in 9 mL serum tubes (Vacuette^®^, Z Trace Elements Serum Clot Activator; Greiner bio-one, Kremsmünster, Austria). Immediately after collection, the samples were refrigerated, transported to the laboratory and centrifuged at 3000 rpm for 10 min to obtain plasma and serum, within 4 h of collection. Triplicate subsamples were stored at −20° pending analysis.

### 2.2. Reagents

All solutions were prepared using ultrapure water of resistance 18 MΩ cm^−1^ (produced using a Milli-Q purification system, Millipore Corp., Bedford, MA). Stock standard solutions of the elements (1000 mg L^−1^) were ultrapure grade: ICP Multi element standard solution IV certiPUR^®^, for B, Ba, Ca, Cd, Co, Cr, Cu, Fe, Li, Mg, Mn, Ni, P, Pb, Sr and Zn determination, and ICP standard certiPUR^®^, for Hg and Se determination, were both purchased from Merck (Poole, U.K.). As, Mo, Sb and U standards were obtained from Panreac (Barcelona, Spain). The samples were acid digested in nitric acid (69%, Hiperpur-Panreac, Barcelona, Spain) and hydrogen peroxide (33% *w*/*v*, Panreac, Barcelona, Spain). The certified reference material (NIST SRM-1598a inorganic constituents in animal serum) used to validate ICP-MS measurements was obtained from the National Institute for Standards and Technology (NIST) (Gaithersburg, MD, USA). Polypropylene tubes used for preparation of samples and standards were soaked in 10% Hyperpur^®^ HNO_3_ for at least 24 h and rinsed with deionised water and dried before use. The sample tubes were tested and found to be free of trace elements.

### 2.3. Sample and Standard Preparation

Aliquots (1 mL) of plasma and serum samples were digested using an acid digestion procedure, as previously described [6]. Briefly, the plasma or serum samples were digested by mixing with 1 mL of concentrated HNO_3_ and 0.5 mL H_2_O_2_ in propylene tubes and leaving at 60 °C for 2 h. The digest thus obtained was diluted by adding 2.5 mL of ultrapure water and centrifuging at 2000 rpm for 5 min. The working calibration standard solutions were prepared daily by appropriate dilution of the previously described multi-elemental standards in 20% (*v*/*v*) nitric acid, and internal standards (20 μg L^−1^ of Ge and Tb) were added online at a flow rate of 40 μL min^−1^. 

### 2.4. Multi-Element Determination

ICP-MS-based multi-element determination was performed in an Agilent 7700x ICP-MS system (Agilent Technologies, Tokyo, Japan) equipped with collision/reaction cell interference reduction technology. The continuous sample introduction system consisted of an autosampler, a Scott double-pass spray chamber (Agilent Technologies, Tokyo Japan), a glass concentric MicroMist nebuliser (Glass Expansion, West Melbourne, Australia), a quartz torch and nickel cones (Agilent Technologies, Tokyo Japan). Elemental concentrations were quantified using MassHunter Work Station Software for ICP-MS (version A.8.01.01 Agilent Technologies, Inc. 2012, Tokyo, Japan). All samples were blank corrected and analysed in triplicate, with Ge and Tb as internal standards. Calibration curves (concentration range, 0.2 to 10.000 µg L^−1^) were constructed daily, by analysis of fresh standard solutions, immediately before analysis of the serum samples. In all cases, linear responses were obtained with zero intercept, correlation coefficients higher than 0.999 and a relative standard deviation (RSD) below 5%.

### 2.5. Quality Control

A quality assurance program was applied during the study (Table 1). Analytical blanks (prepared following exactly the same procedure as for plasma and serum samples) were included in all batches, and the corresponding results were used to calculate the limits of detection (LOD, calculated as 3 times the standard deviation of the blanks); in all of the bovine serum and plasma samples, mineral concentrations were above the LOD. Analytical accuracy was verified by using as a certified reference material (CRM) Animal serum NIST 1598a and spiked samples at the appropriate concentration levels (up to 2–10 times higher than the normal levels in plasma and serum). Overall, good recovery of both the CRM and the spiked samples was achieved. 

In addition, the intra-sample precision (assessed from 10 repetitions of the same sample) and inter-assay precision (evaluated by preparing 10 digest solutions of the same sample on different days) were determined, for both plasma and serum samples.

### 2.6. Data Processing and Statistical Analysis

AII statistical analyses were conducted with SPSS software for Windows (version 21). The standard relative deviation (SRD) was used to evaluate the precision of the analysis of the plasma and serum samples. Differences between mineral concentrations in plasma and serum were checked by *t*-test. Finally, the association between mineral concentrations in plasma and serum was evaluated using the Pearson’s correlation coefficient. 

## 3. Results and Discussion

### 3.1. Intra and Inter-Assay Precision 

The intra-sample precision (assessed from 10 repetitions of the same sample) and inter-assay precision (evaluated by preparing 10 digest solutions of the same sample on different days) of essential and toxic elements determination in plasma and serum samples are shown in Table 2. Overall, acceptable results (<5% for the intra-assay and <10% for the inter-assay study for most elements) were obtained, with only small differences between plasma and serum samples. However, the analytical precision tended to be slightly higher for the plasma samples and most elements, particularly those of interest for assessing the health and nutritional status of the herd (macroelements and trace elements). These findings were expected as possible matrix effects and/or interference in both types of samples were similar and possibly low after the acid digestion. This, together with the low limits of detection and the accuracy of the method (determined from the CRM and spiked samples) (Table 1), guaranteed good analytical quality for both types of samples.

### 3.2. Analysis of Serum and Plasma Paired Samples

The essential and toxic element concentrations in paired plasma and serum samples (*n* = 20) are presented in Table 3. Overall macro and trace elements concentrations were within the adequate range for this animal species [12,13] and toxic element concentrations were very low, reflecting a low level of environmental exposure [1,12].

Within the essential trace elements, the concentrations of Cu (40.9%) and Se (29.9%) were statistically significantly higher in the plasma than in the serum, whereas the opposite was true for Mn (9.9%). Significantly lower concentrations of Cu in serum than in plasma have been described for cattle [10] and other bovid ruminant species such as sheep [14] and goats [15], although this has not been observed in cervid ruminants such as red deer [16] and most other mammals [1]. In bovid ruminants, significant sequestration of Cu occurs during the early stages of the clotting process [17]. It has been estimated that serum Cu concentrations in cattle are, on average, around 3 µmol L^−1^ (190 mg L^−1^) lower than the concentrations in plasma [10]. However, the differences between serum and plasma concentrations vary greatly between individual animals. It has generally been considered that ceruloplasmin (CP) is the primary Cu-containing component of the blood, as it accounts for 70–90% of the total Cu in the plasma [18]. CP is mainly lost during clotting, and other parts of the plasma Cu pool (as the Cu-Zn-containing superoxide dismutase) are not affected [17]. Although the mechanisms involved in the loss of CP during clotting are not known, it has been suggested that the trigger involved may be one of the proteases formed in the clotting cascade. Thus, the protease may activate sialic acid residues or their receptors to induce binding of CP, which may occur through attachment of the sialic acid residues present on CP to receptors on erythrocytes or platelets. Alternatively, CP sequestration may involve the sialic acid residues of platelets, or to a lesser extent the erythrocytes, which contain considerably less sialic acid than platelets [19]. More recent studies indicate that other fractions of the Cu pool apart from CP may be involved [20], although no other mechanisms have been proposed. As far we are aware, the possible effect of clotting on other trace elements, which may explain the significant decrease in Se in the serum found in our study, has not been described in ruminants or other species including humans. In the plasma, Se occurs in the form of selenoprotein-P, glutathione peroxidase and albumin [21], and although Se dependent enzyme glutathione peroxidase is known to play a role in thrombus formation [22,23], we are not aware of the mechanisms whereby loss of Se could occur during the clotting process. However, the fact that the Cu and Se apparently lost during clot formation (estimated as the ratio Cu/Se in serum/plasma) were correlated (*p* < 0.05) may suggest that some Se is lost during the clotting process. This finding deserves further investigation.

Moreover, to the best of our knowledge the apparently higher Mn concentration in the serum than in plasma samples from cattle has not been previously reported. Higher serum concentrations of Fe and Zn have been described in serum samples in humans [24] and are attributed to leakage from the erythrocytes, which makes control of haemolysis essential during blood extraction and laboratory manipulation [24]. Significant release of Zn from platelets, which aggregate or disintegrate during the clotting process induced in order to obtain serum, has also been described [22]. None of the above-described studies evaluated the effect of haemolysis and/or clotting on an apparent increase in Mn concentration in serum. However, most of the Mn in blood is known to be contained within the erythrocytes (accounting for 68% of the total Mn in the blood, [25]) so that even a low level of haemolysis could release a significant amount of Mn to the serum. Although not statistically significant, the Fe concentrations were higher in serum (15%) than in plasma in our study, possibly suggesting that a low level of haemolysis (less than 0.5 g Hb L^−1^; [24] occurred in some samples. This finding, together with the powerful correlation Pearson coefficient (r = 0.890, *p* < 0.001) between the serum/plasma ratios of Fe and Mn concentrations, may indicate some leakage from the erythrocytes as one possible reason for the higher Mn and Fe concentrations in the serum in our study. 

**Figure 1:**
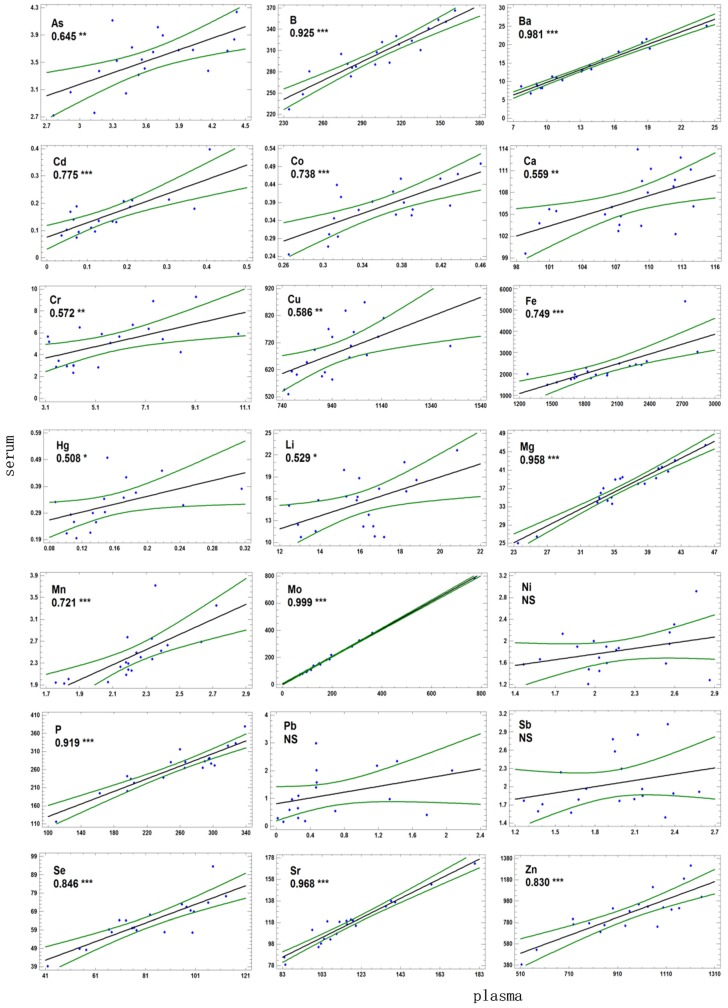
Relationship between the concentrations of essential and toxic elements in plasma and serum (in µg L^−1^, except for Ca, P and Mg in mg L^−1^ ) expressed as coefficient de correlation of Pearson (r) and probability (* *p* < 0.05, ** *p* < 0.01, *** *p* < 0.001).

Statistically significant differences were also observed within the toxic elements, i.e., for Hg and Pb, which were approximately two times more abundant in the serum than in the plasma. Both Pb [26] and Hg [27] have a high affinity for erythrocytes and it is possible that the higher concentrations in serum may be related to leakage from red cells during handling of blood samples. However, the concentrations of both toxic elements were very low in our samples (in the range of 1 µg L^−1^, corresponding to a very low level of environmental exposure, [12]), and no significant correlations were found between the plasma/serum ratio of these elements and those of Mn and Fe (*p* < 0.05) that would support this hypothesis. Another possible explanation is contamination of the tubes during blood collection; however, this would be expected to affect all serum samples to the same extent, increasing the serum concentrations in all samples above background. If this were the case, plasma and serum concentrations would be correlated (Figure 1); however, the Pb concentrations in plasma and serum were not correlated, and those Hg were only very weakly correlated. 

### 3.3. Correlations between Plasma and Serum

The results of the correlation analysis between plasma and serum concentrations are presented in Figure 1. Overall, statistically significant correlations were found for most of the elements (except Ni, Pb and Sb) analysed in our study. 

The association was very powerful for some elements such as Mo (r = 0.999, *p* < 0.001), but was generally high (r > 0.8, *p* < 0.001) for most essential elements of main interest from the point of view of health and nutrition. The variation between serum and plasma concentrations can be explained by considering the variability in the analytical precision (intra and inter-assay precision). The only exception was Ca where only a weak association between serum and plasma was found (r = 0.559, *p* < 0.01); our results are in agreement with previous studies that indicate a significant effect of the anticoagulant on the analysis of Ca, that leads to recommend the determination of Ca in serum [28]. As stated, haemolysis of some serum samples was suspected to have occurred, and for Fe and Mn the correlations between serum and plasma concentration were much lower (r = 0.749 and 0.721 respectively) and were negatively influenced by these samples (Figure 1). When these samples were excluded from the analysis, the correlation was stronger. For Cu, the correlation was lower (r = 0.586, *p* < 0.01), which is in accordance with published data in cattle indicating a weak relationship between Cu in serum and plasma because of the marked variability in the apparent loss of Cu during clotting [10]. On the contrary, for the minor or only occasionally beneficial trace elements (Cr, Li and Ni) and the toxic elements, lower or not significant associations between plasma and serum concentrations were found. All these elements are at very low concentrations in the blood (close to the detection limits) making their analytical quantification highly influenced by the inter-analysis variation [6,12]. It is also possible that some of these elements could have an affinity to the erythrocytes (as previously stated for Hg and Pb) so that some degree of haemolysis could exert a high influence on the correlation analysis. 

## 4. Conclusions

Our findings indicate that both plasma and serum samples appear suitable and interchangeable for the determination of most of the essential and toxic elements in blood in cattle. The only exceptions seem to be Cu and Se, which were detected at significantly lower levels in the serum than in plasma. Although this has been described for Cu in bovine ruminants, the finding in relation to Se deserves further research in other species. Finally, special attention should be paid to preventing haemolysis of blood samples during collection and processing, particularly of serum, to prevent overestimation of elements present at high concentrations (i.e., Fe, Mn and Zn) inside the erythrocytes.

## Figures and Tables

**Table 1:** Result of the analytical quality control program for plasma and serum samples.

Element	DL	Animal Serum NIST 1598a		Spiked Samples (%)	
	(µg L^−1^)	Certified Value (µg L^−1^) ^1^	Recovery (%)	Plasma	Serum
As	0.002	(0.3) ^2^	ND	93.8 ± 7.4	84.2 ± 6.5
B	0.001	--		93.1 ± 5.2	95.8 ± 13.1
Ba	0.010	--		91.0 ± 4.9	83.9 ± 4.1
Ca	0.020	96 ± 7	95	101.4 ± 3.8	99.8 ± 5.2
Cd	0.011	0.048 ± 0.004	91	96.1 ± 5.7	99.1 ± 10.1
Co	0.008	1.24 ± 0.07	93	88.8 ± 3.2	91.1 ± 1.7
Cr	0.093	0.33 ± 0.08	ND	91.5 ± 8.2	96.4 ± 12.0
Cu	0.130	1580 ± 90	95	91.1 ± 5.7	96.1 ± 5.7
Fe	0.147	1680 ± 60	102	91.5 ± 8.2	95.8 ± 13.1
Hg	0.050	0.32 ± 0.19	94	89.1 ± 8.4	84.2 ± 3.2
Li	0.141	--		88.1 ± 9.4	105.1 ± 11.0
Mg	0.050	--		92.6 ± 5.2	96.1 ± 5.7
Mn	0.014	1.78 ± 0.33	103	91.5 ± 1.2	88.8 ± 4.4
Mo	0.089	5.5 ± 1.0	98	96.1 ± 5.7	86.1 ± 8.7
Ni	0.112	0.94 ± 0.18	97	97.1 ± 6.9	106.1 ± 5.7
P	0.017	(140)	99	96.1 ± 5.7	101.3 ± 2.6
Pb	0.022	--		89.4 ± 11.9	89.4 ± 5.2
Sb	0.001	1.00 ± 0.15	105	93.1 ± 5.7	96.1 ± 5.7
Se	0.023	134.4 ± 5.8	102	99.1 ± 7.7	98.1 ± 5.2
Sr	0.015	--		105.4 ± 4.8	99.5 ± 1.2
Zn	0.005	880 ± 24	98	91.5 ± 6.5	95.6 ± 6.7

^1^ except for Ca, Mg and P that are expressed in mg L^−1^; ^2^ in brackets only indicative values; ND: not detected; DL: detection limit.

**Table 2:** Results of the intra and inter-assay precision (expressed as relative standard deviation (RSD)) for plasma and serum samples.

Element	*Intra*-Assay (*n* = 10)		*Inter*-Assay (*n* = 10)	
	Plasma	Serum	Plasma	Serum
As	4.54	5.33	4.36	4.50
B	2.46	2.88	4.19	7.17
Ba	2.55	2.76	5.11	4.51
Ca	1.79	1.93	2.46	2.59
Cd	6.81	6.04	7.49	7.64
Co	2.14	1.89	3.84	3.67
Cr	3.36	4.59	8.22	8.96
Cu	1.83	2.84	2.35	5.24
Fe	1.69	2.20	3.70	4.07
Hg	7.98	8.24	8.33	9.67
Li	4.92	6.84	6.11	6.64
Mg	2.06	2.46	4.57	4.72
Mn	2.91	2.44	2.77	4.74
Mo	1.07	1.14	1.97	2.48
Ni	3.93	3.96	6.76	7.10
P	2.67	3.20	2.96	3.32
Pb	2.46	4.97	5.66	6.79
Sb	2.96	3.27	3.86	3.20
Se	2.86	3.22	3.09	3.28
Sr	2.26	1.87	3.20	2.87
Zn	1.64	2.23	2.10	2.57

**Table 3:** Comparison of essential and toxic element concentrations in paired (*n* = 20) bovine serum and plasma samples. All elements expressed in µg L^−1^, except Ca, Mg and P that are in mg L^−1^.

Element	Plasma			Serum			*p*-Value
	Mean ± SE	Median	Range	Mean ± SE	Median	Range	
As	3.63 ± 0.11	3.57	2.76–4.42	3.53 ± 0.09	3.59	2.72–4.24	0.492
B	302 ± 8	303	234–361	305 ± 8	306	227–366	0.797
Ba	13.6 ± 1.05	13.1	7.69–24.3	13.5 ± 1.20	12.8	6.74–25.2	0.943
Ca	108 ± 1	109	99–114	107 ± 1	106	100–114	0.306
Cd	0.158 ± 0.025	0.129	0.036–0.407	0.169 ± 0.020	0.140	0.074–0.397	0.715
Co	0.361 ± 0.012	0.371	0.265–0.460	0.379 ± 0.015	0.376	0.245–0.498	0.367
Cr	5.72 ± 0.49	5.28	3.19–10.8	5.06 ± 0.44	5.30	2.34–9.29	0.326
Cu	965 ± 36	941	748–1418	685 ± 21	684	530–869	0.001
Fe	1969 ± 90	1882	1274–2840	2263 ± 186	2003	1457–5411	0.852
Hg	0.153 ± 0.012	0.138	0.087–0.316	0.321 ± 0.021	0.319	0.195–0.497	0.001
Li	16.0 ± 0.5	16.0	12.5–20.9	15.5 ± 0.8	15.8	10.8–22.7	0.549
Mg	36.0 ± 1.2	35.4	23.5–45.9	37.1 ± 1.1	38.0	25.0–46.4	0.510
Mn	2.22 ± 0.05	2.20	1.76–2.72	2.44 ± 0.103	2.38	1.93–3.72	0.026
Mo	185 ± 36	135	69–773	191 ± 36	144	71–784	0.911
Ni	2.16 ± 0.09	2.09	1.46–2.86	1.88 ± 0.11	1.85	1.21–3.04	0.051
P	255 ± 13	266	110–339	265 ± 13	274	116–379	0.590
Pb	0.659 ± 0.146	0.457	0.014–2.068	1.188 ± 0.200	0.970	0.154–3.050	0.043
Sb	1.93 ± 0.09	1.98	1.27–2.58	2.02 ± 0.10	1.87	1.49–3.03	0.484
Sr	118 ± 5	114	84–181	117 ± 5	117	79–173	0.966
Se	82.6 ± 4.4	80.1	41.9–113.2	63.6 ± 2.6	62.2	39.3–93.6	0.001
Zn	952 ± 47	978	514–1256	862 ± 47	892	387–1313	0.185
